# Prolonged Gastric Transit Time in Small-Bowel Capsule Endoscopy: Which Patients Are at Risk and What Are the Implications?

**DOI:** 10.5152/tjg.2023.22191

**Published:** 2023-03-01

**Authors:** Marta Freitas, Vítor Macedo Silva, Sofia Xavier, Pedro Boal Carvalho, Bruno Rosa, Maria João Moreira, José Cotter

**Affiliations:** 1 Department of Gastroenterology, Hospital da Senhora da Oliveira, Guimarães, Portugal; 2 Life and Health Sciences Research Institute (ICVS), University of Minho Faculty of Medicine, Braga, Portugal; 3 ICVS/3B’s, PT Government Associate Laboratory, Braga, Guimarães, Portugal

**Keywords:** Capsule endoscopy, gastric transit time, gastrointestinal transit, incomplete examination, risk factors

## Abstract

**Background:**

Prolonged gastric transit time is a commonly described cause for incomplete capsule endoscopy examination. This study aimed to evaluate the prevalence and identify risk factors for prolonged gastric transit time and to assess its impact on the rate of incomplete examinations.

**Methods:**

This is a retrospective study including patients undergoing small-bowel capsule endoscopy between January 2014 and August 2020. Patients with prolonged gastric transit time were consecutively included and patients without prolonged gastric transit time were randomized (controls) in a 1:2 ratio. Prolonged gastric transit time was defined as small-bowel capsule endoscopy remaining in the stomach for more than 1 hour, as checked with the routine use of the real-time viewer, requiring an intervention such as prokinetic administration and/or endoscopically assisted capsule delivery into the duodenum.

**Results:**

Prolonged gastric transit time occurred in 45/957 patients (prevalence 4.7%). Both groups were similar regarding small-bowel capsule endoscopy indication and inpatient status. The mean small-bowel transit was similar between groups (4 hours 48 minutes ± 2 hours 11 minutes vs. 4 hours 38 minutes ± 1 hour 36 minutes; *P*  = .74). Prolonged gastric transit time group did not have a significant higher rate of incomplete exams (*P*  = .44) but presented more frequently with inadequate small-bowel preparation (*P*  < .001). Older age (*P*  = .046), female sex (*P*  = .004), diabetes (*P*  = .03), and psychotropic medication use (*P*  = .02) were risk factors for prolonged gastric transit time. In multivariate analysis, female sex (odds ratio: 4.0; *P*  = .002) and psychotropic medication use (OR: 4.6; *P*  = .003) were predictors of prolonged gastric transit time.

**Conclusion:**

Prolonged gastric transit time was not associated with a higher rate of incomplete exams in our cohort but was associated with higher rate of inadequate small-bowel preparation. Female sex and psychotropic medication use were independent risk factors for prolonged gastric transit time.

**Main Points**

- Female sex and psychotropic medications were independent risk factors for prolonged gastric transit time.
- Prolonged gastric transit time was not associated with a higher rate of incomplete exams.
- Real-time monitoring of capsule passage to the duodenum prevents incomplete exams.
- Early preventive interventions such as prokinetic administration and/or endoscopically assisted capsule delivery prevent incomplete exams.

## Introduction

Small-bowel capsule endoscopy (SBCE) is a non-invasive procedure, introduced in clinical practice in 2001, that has undeniably revolutionized the diagnosis and management of small-bowel pathology.^1^ An important limitation of SBCE is the occurrence of incomplete examination, considered when the capsule does not reach the cecum within the battery recording time, that is of approximately 8 hours.^2^ The frequency of incomplete examination reported in the literature is not negligible, occurring in up to 20%-30% of patients^2-4^ and can compromise the accuracy of the exam, especially in the distal segments of the small bowel, potentially leading to the need for further examinations and increased costs.^2,5^ Prolonged gastric transit time (PGTT) was commonly described as a cause of incomplete SBCE examination^2,6^ and is believed to account for 30% of incomplete procedures.^7^ Prolonged gastric transit time corresponds to delayed gastric transit resulting in SBCE remaining in the stomach for more than 30-120 minutes.^2,4,8,9^ In some reports, the rates of PGTT ranged between 0% and 5%.^8,10,11^ Certain conditions have been identified as risk factors for PGTT such as older age,^11^ female sex,^12^ lower body mass index (BMI),^12,13^ ongoing hospitalization,^8^ diabetes,^14^ hypothyroidism,^11^ and lower physical activity during examination.^13^ Surprisingly, gastroparesis has not been correlated with PGTT.^9^

Approaches aimed at overcoming delayed capsule gastric emptying to improve visualization and completion of SBCE examination to date include the development of an adaptable frame rate,^15,16^ longer battery life,^17^ positional changes,^18^ prokinetics administration,^5,19,20^ and use of a 3-dimensional localization method.^12,21,22^ Cotter et al^5^ showed that the use of real-time viewer and selective administration of domperidone to patients with delayed gastric passage of the capsule significantly reduce incomplete examinations from 15.6% to 3.7%.

Remarkably, PGTT on SBCE has not very often been discussed in the literature and most of the studies focused on predictors for incomplete SBCE examinations and not specifically for PGTT. However, it is important before SBCE examination to identify factors that may lead to PGTT of the capsule to reduce incomplete examinations and improve the cost-effectiveness of the procedure.

The aim of this study was to evaluate the prevalence and identify risk factors for PGTT and to assess its impact on the rate of incomplete examinations.

## Materials and Methods

This study was retrospective and descriptive without prospective interventions. All patients received current standard of care, without experimental intervention. All the collected data were anonymized. The study protocol conforms to the ethical guidelines of the 1975 Declaration of Helsinki as reflected in a priori approval by the institution’s ethics committee. All patients were given standard instructions and submitted written informed consent before the SBCE procedure.

### Patients and Definitions

We performed a case–control, retrospective, single-center study including all consecutive patients undergoing SBCE in the Gastroenterology Department of a university-affiliated Hospital, between January 2014 and August 2020. All patients with PGTT in SBCE in the given study period were consecutively included (cases). Patients without PGTT were randomized (controls), using an automatic number randomization software (Winpepi), to attain a 1:2 cases to controls ratio.

Small-bowel capsule endoscopy was performed for various indications such as overt gastrointestinal (GI) bleeding, occult GI bleeding (anemia and/or positive fecal occult blood test), and suspected and established Crohn’s disease. We excluded patients under 18 years and with previous gastric surgery.

Demographic and clinical data were collected by reviewing medical records and included patients’ age, gender, smoking habits, comorbidities (including diabetes, arterial hypertension, chronic kidney disease, cirrhosis, heart failure, cerebrovascular disease, hypothyroidism, depression, obesity (BMI ≥ 30 kg/m^2^), psychotropic medication(s), and inpatient status.

### Small-Bowel Capsule Endoscopy Procedure and Review

The device used for the SBCE procedure was PillCam SB3® (Medtronic, Dublin, Ireland). Patients were given instructions to start a clear liquid diet 24 hours prior to the exam and to fast for 12 hours before the exam^23^ and were asked to stop taking oral iron supplements 5 days before the examination. Patients were allowed to drink clear liquids 2 hours after SBCE ingestion (if passage into the small bowel has been confirmed with the real-time viewer function of the portable Data Recorder DR3) and to eat solid food 4 hours later.^23^

The SBCE videos were reviewed by gastroenterologists experienced in capsule reading (>500 reviews), using the RAPID Reader® software (Medtronic). The reading was performed at a maximum speed of 10 frames per second in a single-view mode, as previously recommended.^3^

Endoscopic data were collected by reviewing SBCE examination records and included gastric transit time, small-bowel transit time, small-bowel preparation quality, relevant endoscopic findings defined as the presence of P2 lesions (high bleeding potential, such as angioectasias, ulcers, tumors, or varices), according to Saurin *et al* classification,^24^ and incidence of incomplete exams.

Gastric transit time was defined as the time in which SBCE remained in the stomach, determined after selecting the first gastric and duodenal landmarks in the RAPID Reader® software. Small-bowel transit was defined as the time in which SBCE remained in the small bowel, determined after selecting the first duodenal and cecal landmarks, or alternatively the last image of the small bowel if the capsule did not reach the cecum within recording time.

Prolonged gastric transit time was defined as SBCE remaining in the stomach for more than 1 hour due to delayed gastric transit, as checked with the systematic use of the real-time viewer. In these cases, 10 mg of domperidone was administered orally and the location of the capsule was rechecked after 30 minutes with the real-time viewer. If it still remained in the stomach, an additional dose of 10 mg of domperidone was administered orally, and after another 30 minutes, the location of the capsule was rechecked; then if still in the stomach, the capsule was placed directly in the duodenum by upper endoscopy using a basket or snare.

An incomplete examination was defined when SBCE did not reach the cecum within the battery time.

To assess the quality of small-bowel cleansing, we followed a method similar to other publications,^25,26^ by evaluating the proportion of the small-bowel mucosa during which mucosal observation was adequate, without any liquid, bubbles, or debris. Small-bowel preparation was classified as excellent, if an ideal visualization was achieved in over 90% of the recording time; good, if the mucosa was in perfect condition in 75%-90% of the recording time, with some fluid or debris which did not seem to compromise the overall quality of the examination; fair, if only 50%-75% of the mucosa was under perfect conditions for observation of the recording time, with the presence of enough fluid, bubbles, or debris to preclude a completely reliable examination; and poor, if only <50% of the mucosa could be perceived during the recording time, with significant amounts of fluid, bubbles, or debris compromising the interpretation of the examination. We considered that the cleansing was inadequate if a fair or poor preparation was verified with less than 75% of the small-bowel mucosa in perfect condition for visualization.

### Statistical Analysis

Statistical analysis was performed with Statistical Package for the Social Sciences® software version 24.0 (IBM, Armonk, NY, USA). Categorical variables are presented as frequencies and percentages and continuous variables as mean and standard deviation. Categorical variables were compared using *χ* ^2^-test or Fisher’s exact test (2-tailed) as appropriate. Continuous variables were compared using Student’s *t*-test. A multivariate analysis using a binary logistic regression was performed to evaluate predictive factors for PGTT. The selection of the variables to include in the multivariate analysis was performed by considering those with statistical significance in the univariate analysis. Statistical significance of univariate and multivariate analysis was defined for *P* <.05.

## Results

### Demographic and Clinical Data

A total of 975 SBCE procedures were performed in our department during the study period. Eighteen patients were excluded from the study; 13 patients had previous gastric surgery and 5 patients had dysphagia, thus the capsule was introduced directly into the duodenum through upper endoscopy using a capsule endoscope delivery device—the AdvanCE® (US Endoscopy, Mentor, OH, USA). The remaining 957 patients were included in the study analysis. Prolonged gastric transit time occurred in 4.7% of the patients (45/957 patients). We randomly selected a total of 90 participants as controls among the 912 eligible patients without PGTT, as explained in the methods.

Baseline demographic and clinical characteristics of patients with PGTT are listed in Table 1. Patients had a mean age of 52 ± 20 years and 42.2% (*n* = 19) were female. The most common comorbidity was arterial hypertension (28.9%, *n*  = 13), followed by diabetes and depression (24.4%, *n*  = 11). Three patients (6.7%) had smoking habits and 13 patients (28.9%) were under psychotropic medication. Concerning inpatient status, 3 patients (6.7%) were hospitalized when SBCE examination was performed. Regarding SBCE indication, the majority of patients, 51.1% (*n* = 23), performed SBCE for iron-deficiency anemia, 37.8% (*n* = 17) for suspected Crohn’s disease, 8.9% (*n* = 4) for established Crohn’s disease, and 2.2% (*n* = 1) for overt GI bleeding.

### Small-Bowel Capsule Endoscopy Data

Small-bowel capsule endoscopy data of patients with PGTT are included in Table 1. Patients with PGTT had a mean gastric transit time of 2 hours 34 minutes ± 40 minutes and a mean small-bowel transit time of 4 hours 48 minutes ± 2 hours 11 minutes. Totally 7 patients (15.6%) had inadequate small-bowel preparation Four patients (8.9%) had an incomplete SBCE, 1 of which with suspected Crohn’s disease (CD) had a stenosis, but all patients remained asymptomatic, and a routine abdominal radiography performed after 14 days excluded the presence of the capsule within the bowel. Regarding relevant endoscopic findings, 15 patients (33.3%) had P2 lesions in SBCE examination.

### Risk Factors for Prolonged Gastric Transit Time

Univariate analysis comparing the characteristics of patients with PGTT with controls is presented in Table 2. Both groups were similar regarding inpatient status (*P*  = .33) and SBCE indication: iron-deficiency anemia (*P*  = .09), suspected Crohn’s disease (*P*  = .05), established Crohn’s disease (*P*  = .65), and obscure GI bleeding (*P*  = 1.0). The mean gastric transit was higher in PGTT group (2 hours 34 minutes ± 40 minutes vs. 39 minutes ± 41 minutes; *P*  < .001). The mean small-bowel transit was similar between groups (4 hours 48 minutes ± 2 hours 11 minutes vs. 4 hours 38 minutes ± 1 hour 36 minutes; *P*  = .74). Prolonged gastric transit time group did not have a significant higher rate of incomplete exams (*P*  = .44) but presented more frequently with inadequate small-bowel preparation (*P*  < .001). There was no difference regarding the diagnosis of P2 lesions in SBCE between groups (*P*  = .22). Older age (*P*  = .046), female sex (*P*  = .004), diabetes (*P*  = .03), and psychotropic medication use (*P*  = .02) were risk factors for PGTT. There was no difference between groups in the frequency of arterial hypertension (*P*  = .89), hypothyroidism (*P*  = .33), chronic kidney disease (*P*  = .69), cirrhosis (*P*  = 1.0), heart failure (*P*  = .08), cerebrovascular disease (*P*  = .67), depression (*P*  = .07), obesity (*P*  = .44), and smoking habits (*P*  = .33).

In multivariate analysis, female sex (*P*  = .002) and psychotropic medication use (*P*  = .003) were predictors of PGTT. Female sex and psychotropic medication use were associated with a 4.0- and 4.6-fold increased risk of PGTT, respectively (Table 3).

## Discussion

A complete SBCE examination depends upon normal GI motility to propel the device through the esophagus, stomach, and small intestine during the battery lifespan. Delayed gastric passage of the capsule has been consistently reported as a major factor leading to incomplete SBCE,^2,4,6^ which may compromise the accuracy of the exam, increasing the need for further examinations and the costs.^2,5^

Our study reported a prevalence of PGTT of 4.7%, similar to previous studies.^4,8,10,11^

Regarding risk factors for PGTT, the data are variable.^8,9,11-14^ We found that older age, female sex, diabetes, and psychotropic medication use were risk factors for PGTT, which is in line with some reports in the literature,^11,12,14^ although other studies found no correlation between PGTT and age,^8,27^ gender,^11,27^ and diabetes.^9,11,12^ Patients with diabetes frequently have delayed gastric emptying^28,29^ and even gastroparesis.^30^ The main pathogenetic factors are vagal autonomic neuropathy, interstitial cells of Cajal pathology, and hyperglycemia.^30^ Lower physical activity during SBCE examination was previously correlated with PGTT.^13^ Regarding the retrospective nature of our study, this variable was not possible to include. Hypothyroidism has been previously identified as risk factor for PGTT,^11^ but we did not find this association. Due to the retrospective nature of the study, we did not have laboratory analysis with thyroid profile at the same time as the capsule endoscopy was performed. Prospective studies are required to confirm the role of thyroid pathology in the risk of PGTT.

Ongoing hospitalization was reported as a cause of PGTT and incomplete SBCE examination. It was hypothesized that the supine position of the patient during the recording and perhaps the stress induced by acute illness were possible explanations, and it was suggested that these patients may benefit from administration of a prokinetic agent.^8,9^ However, in our cohort, we found no association between ongoing hospitalization and PGTT.

Some approaches emerged to overcome PGTT in order to improve visualization and completion of SBCE examination and include the development of a faster adaptable frame rate,^15,16^ longer battery life,^17^ positional changes,^18^ prokinetics administration,^5,19,20^ and use of a 3-dimensional localization method.^12,21,22^ In our center, we previously showed that the use of real-time viewer and selective administration of domperidone to patients with delayed gastric passage of the SBCE significantly reduce incomplete examinations.^5^ Accordingly, European Society of Gastrointestinal Endoscopy (ESGE) recommendations advocate the use of a real-time viewer, particularly in patients at risk of delayed gastric emptying, to guide appropriate preventive intervention such as prokinetic administration and/or endoscopically assisted capsule delivery into the duodenum.^23^ Furthermore, these measures were considered a quality indicator for SBCE.^31^ In our study, PGTT group did not have a significantly higher rate of incomplete exams. We think that it occurred, because as recommended by European guidelines,^23^ we routinely perform real-time monitoring of the capsule passage, and if SBCE remains in the stomach for more than 1 hour, we take early preventive interventions such as prokinetic administration and/or endoscopically assisted capsule delivery, optimizing the SBCE examination time.

The effects of routine use of prokinetics, such as metoclopramide and erythromycin, to enhance gastric transit were inconsistent, reflecting the variety in study designs which included different administration routes (oral or parenteral) and timing of administration of prokinetics.^6,19,20^ A meta-analysis of 4 randomized controlled trials evaluating the role of prokinetics in SBCE showed that prokinetic use alone was ineffective in increasing SBCE completion rates.^19^ On the other hand, patients with increased risk for an incomplete SBCE study, with previous history of abdominal surgery, delayed gastric emptying, diabetic neuropathy, severe hypothyroidism, use of psychotropic drugs, may benefit from the administration of prokinetics (metoclopramide or domperidone), when the capsule remains in the stomach for more than 30‐60 minutes as confirmed by real-time monitoring.^19^

Although PGTT group did not have a significantly higher rate of incomplete exams, it presented more frequently with inadequate small-bowel preparation. However, this probably did not affect SBCE diagnostic yield since there were no differences regarding the diagnosis of P2 lesions between patients with PGTT versus without PGTT. Westerhof et al^32^ found that a faster passage of the capsule through the small bowel has been associated with lower diagnostic yield of SBCE, showing that delayed small-bowel transit may be more determinant factor in SBCE diagnostic yield than the gastric transit time. We further analyzed whether a PGTT was related to longer small-bowel transit time and found no correlation, as previously described.^2^

Our study has few limitations such as its retrospective nature, which depends on clinical records, and its small sample size that is justified by the fact that PGTT is not a frequent event. Despite these limitations, we believe that our study is relevant, being valuable in the optimization of the SBCE procedure. Prospective, multicentric, larger studies are required to confirm our findings and to understand the mechanisms why older age, female sex, diabetes, and psychotropic medications increase the risk of PGTT.

In conclusion, in our cohort, PGTT was not associated with a higher rate of incomplete exams, and we believe this is related to the fact that we routinely perform real-time monitoring of the capsule passage to the duodenum and early preventive interventions such as prokinetic administration and/or endoscopically assisted capsule delivery. Therefore, we suggest that efforts should be made to adopt this strategy in all SBCE examinations, even more importantly in those with risk factors for PGTT, in order to optimize the SBCE examination time and to prevent a higher rate of incomplete exams.

## Figures and Tables

**Table 1.** Baseline Demographic and Clinical Characteristics of Patients with PGTT on SBCE Examination

Age, mean ± SD, years	52 ± 20
Gender, female, n (%)	19 (42.2)
Smoking, n (%)	3 (6.7)
Comorbidities, n (%)	
Diabetes	11 (24.4)
Arterial hypertension	13 (28.9)
Hypothyroidism	3 (6.7)
Chronic kidney disease	3 (6.7)
Cirrhosis	1 (2.2)
Heart failure	6 (13.3)
Cerebrovascular disease	1 (2.2)
Depression	11 (24.4)
Obesity (BMI ≥30 kg/m^2^)	4 (8.9)
Psychotropic medication(s), n (%)	13 (28.9)
Inpatient status, n (%)	3 (6.7)
SBCE indication, n (%)	
Iron-deficiency anemia	23 (51.1)
Suspected Crohn’s disease	17 (37.8)
Established Crohn’s disease	4 (8.9)
Obscure GI bleeding	1 (2.2)
SBCE data	
Gastric transit time, mean ± SD	2 hours 34 min ± 40 min
Small-bowel transit time, mean ± SD	4 hours 48 min ± 2 hours 11 min
Incomplete exams, n (%)	4 (8.9)
Inadequate preparation, n (%)	7 (15.6)
P2 lesions, n (%)	15 (33.3)

PGTT, prolonged gastric transit time; SBCE, small-bowel capsule endoscopy; SD, standard deviation; BMI, body mass index; GI, gastrointestinal; min, minutes.

**Table 2.** Univariate Analysis Comparing Characteristics of Patients with PGTT with Controls

	PGTT Group	Control Group	*P*
Age, mean ± SD, years	52±20	45±18	.046^*^
Gender, female, n (%)	19 (42.2)	17 (18.9)	.004^*^
Smoking, n (%)	3 (6.7)	2 (2.2)	.33
Comorbidities, n (%)			
Diabetes	11 (24.4)	9 (10.0)	.03^*^
Arterial hypertension	13 (28.9)	25 (27.8)	.89
Hypothyroidism	3 (6.7)	2 (2.2)	.33
Chronic kidney disease	3 (6.7)	4 (4.4)	.69
Cirrhosis	1 (2.2)	2 (2.2)	1.0
Heart failure	6 (13.3)	4 (4.4)	.08
Cerebrovascular disease	1 (2.2)	4 (4.4)	.67
Depression	11 (24.4)	11 (12.2)	.07
Obesity (BMI ≥30 kg/m^2^)	4 (8.9)	4 (4.4)	.44
Psychotropic medication(s), n (%)	13 (28.9)	11 (12.2)	.02^*^
Inpatient status, n (%)	3 (6.7)	2 (2.2)	.33
SBCE indication, n (%)			
Iron-deficiency anemia	23 (51.1)	32 (35.6)	.09
Suspected Crohn’s disease	17 (37.8)	50 (55.6)	.05
Established Crohn’s disease	4 (8.9)	6 (6.7)	.65
Obscure GI bleeding	1 (2.2)	2 (2.2)	1.0
SBCE data			
Gastric transit time, mean ± SD	2 hours 34 min ± 40 min	39 min ± 41 min	<.001^*^
Small-bowel transit time, mean ± SD	4 hours 48 min ± 2 hours 11 min	4 hours 38 min ± 1 hour 36 min	.74
Incomplete exams, n (%)	4 (8.9)	4 (4.4)	.44
Inadequate preparation, n (%)	7 (15.6)	0	<.001^*^
P2 lesions, n (%)	15 (33.3)	40 (44.4)	.22

^*^ Statistically significant *P* values.

PGTT, prolonged gastric transit time; SD, standard deviation; BMI, body mass index; SBCE, small-bowel capsule endoscopy; GI, gastrointestinal; min, minutes.

*P* <.05

**Table 3.** Multivariate Analysis of PGTT Risk Factors.

	*P*	OR	95% CI
Age	.37	1.0	0.99-1.04
Female sex	.002^*^	4.0	1.67-9.81
Diabetes	.29	1.9	0.57-6.39
Psychotropic medication(s)	.003^*^	4.6	1.68-12.5

^*^ Statistically significant* P* values.

PGTT, prolonged gastric transit time; OR, odds ratio; CI, confidence interval.

*P* <.05
